# Chihuahuan Propolis as a Non-Antibiotic Intracanal Agent Against *Enterococcus faecalis*: An In Vitro Intratubular Study

**DOI:** 10.3390/microorganisms14040774

**Published:** 2026-03-28

**Authors:** Hilda Natalia Quintana-Pérez, Grissel Guadalupe Orozco-Molina, Carlos Esteban Villegas-Mercado, Sandra Aidé Santana-Delgado, Adolfo Gonzalez-Acosta, Javier Iván Martínez-Hernández, Raquel Duarte-Rico, Lydia Paulina Loya-Hernández, América Susana Mares-García, Claudia Alejandra Hernández-Escobar, Abigailt Flores-Ledesma, Alejandro Romo Chacón, Mercedes Bermúdez, Juan Antonio Arreguin-Cano

**Affiliations:** 1 Faculty of Dentistry, Autonomous University of Chihuahua, Chihuahua 31000, Mexico; p376143@uach.mx (H.N.Q.-P.); ggorozco@uach.mx (G.G.O.-M.); cmercado@uach.mx (C.E.V.-M.); ssantana@uach.mx (S.A.S.-D.); adgonzalez@uach.mx (A.G.-A.); ivmartinez@uach.mx (J.I.M.-H.); raduarte@uach.mx (R.D.-R.); lploya@uach.mx (L.P.L.-H.); 2 Center for Research in Advanced Materials, S.C. (CIMAV), Chihuahua 31136, Mexico; america.mares@cimav.edu.mx (A.S.M.-G.); claudia.hernandez@cimav.edu.mx (C.A.H.-E.); 3 Dental Materials and Biomaterials Laboratory, Faculty of Stomatology, Meritorious Autonomous University of Puebla, Puebla 72570, Mexico; abigailt.flores@correo.buap.mx; 4 Centro de Investigación en Alimentación y Desarrollo, A.C. Unidad Cuauhtémoc, Chihuahua 31570, Mexico; archacon13@ciad.mx

**Keywords:** propolis, *Enterococcus faecalis*, triple antibiotic paste, dentin tubules, intracanal medicament, biofilm, endodontic infection, antimicrobial activity

## Abstract

Persistent endodontic infections are frequently associated with *Enterococcus faecalis*, a microorganism capable of penetrating dentinal tubules and surviving conventional disinfection procedures. This in vitro study evaluated the antimicrobial activity of Chihuahuan propolis against *E. faecalis* using planktonic and intratubular infection models. Propolis extract was tested at concentrations of 15, 35, and 70 mg/mL and compared with triple antibiotic paste (TAP) as a clinically relevant intracanal medicament. Antimicrobial efficacy was assessed by disk diffusion, minimum inhibitory concentration (MIC), colony-forming unit (CFU) reduction in infected dentinal tubules, and scanning electron microscopy (SEM). Chihuahuan propolis exhibited concentration-dependent antimicrobial activity, with a MIC of 17.5 mg/mL. In the intratubular model, propolis at 70 mg/mL achieved a CFU reduction comparable to TAP after seven days of application. SEM analysis confirmed a marked reduction of bacterial colonization within dentinal tubules. Within the limitations of this in vitro, monoespecies model, Chihuahuan propolis demonstrated antimicrobial efficacy against *E. faecalis* comparable to TAP, supporting its further investigation as a potential non-antibiotic intracanal medicament.

## 1. Introduction

Persistent endodontic infections are a major cause of root canal treatment failure and are often associated with microbial survival within anatomically complex areas and dentin tubules [1]. *Enterococcus faecalis* is the most frequently isolated species in persistent and secondary infections and can form biofilms, survive under nutrient-poor conditions, tolerate high pH, and invade dentin tubules, making it particularly difficult to eradicate [2,3]. Intracanal medicaments such as calcium hydroxide and triple antibiotic paste (TAP) are used to complement chemomechanical preparation and reduce the residual microbial load [4]. Calcium hydroxide has traditionally been considered the gold standard but shows limited effectiveness against *E. faecalis*, difficulties in apical reach, incomplete removal from the canal system, and potential periapical irritation [5]. The TAP, composed of metronidazole, ciprofloxacin, and minocycline, provides broad-spectrum activity and high efficacy in persistent infections, but is associated with dentin demineralization, reduced microhardness, tooth discoloration, hypersensitivity reactions, and concerns about antimicrobial resistance [6].

Propolis is a resinous material produced by honeybees from plant exudates and has been used since antiquity for its antimicrobial, anti-inflammatory, antioxidant, and wound healing properties [7]. Its composition varies according to geographic and botanical origin but typically includes resins, waxes, essential oils, and a rich profile of polyphenols, particularly flavonoids (such as quercetin, galangin, chrysin, and pinocembrin) and phenolic acids (including caffeic, p-coumaric, and ferulic acids) [8]. In this sense, the antibacterial activity of propolis is primarily attributed to the synergistic action of these bioactive compounds. Flavonoids exert their antimicrobial effects by disrupting the bacterial cytoplasmic membrane, inhibiting nucleic acid (DNA and RNA) synthesis, and interfering with energy metabolism and bacterial motility [9]. Concurrently, phenolic acids alter cell membrane permeability, inhibit ATP production, and bind to essential metabolic enzymes, ultimately triggering bacterial cell death [10]. In the same sense, these compounds impair biofilm formation and disrupt ion channels, multi-target mechanisms that are particularly crucial for eradicating persistent, biofilm-forming pathogens like *E. faecalis* within the nutrient-deprived environment of dentinal tubules [2]. Propolis has shown antibacterial and antifungal activity and has been proposed as an irrigant and intracanal medicament, with some studies indicating greater activity than calcium hydroxide and TAP against *E. faecalis* and *C. albicans*, as well as lower cytotoxicity on periodontal ligament cells, fibroblasts, and pulp tissue [11,12].

However, the biological activity of propolis is heavily influenced by its geographic and botanical origin, so results from propolis in different regions cannot be directly applied. In the Chihuahuan Desert of northern Mexico, where locally produced propolis reflects a seasonal and unique flora that may give it a distinctive antimicrobial profile, its potential as an endodontic intracanal medicament has been not totally elucidated.

This in vitro study aimed to assess the antimicrobial effect of propolis from Chihuahua against *E. faecalis* in dentin tubules and to compare its effectiveness with TAP as an intracanal medicament.

## 2. Materials and Methods

### 2.1. Study Design and Sample

A prospective, longitudinal, comparative, experimental in vitro study was conducted on 25 single-rooted human teeth with fully formed apices and straight root canals, obtained for orthodontic or periodontal reasons. The efficacy of propolis from Chihuahua as an intracanal medicament against *E. faecalis* (ATCC 29212) was evaluated in a persistent endodontic infection model using MIC determination and SEM analysis. Planktonic models evaluated free-floating bacterial cells, whereas intratubular models assessed bacterial survival within dentinal tubules, better simulating persistent endodontic infections.

### 2.2. Propolis Sample and Characterization

Chihuahua propolis was collected from a bee farm in Cuauhtémoc, Chihuahua, Mexico, with the following geographical coordinates: 28°24′18″ N (North Latitude) and 106°52′00″ W (West Longitude), at an elevation of 2073 m above sea level. No permission was required to collect the propolis. The Chihuahuan propolis sample was provided by the Food and Development Research Center (CIAD), Cuauhtémoc Unit, Chihuahua.

Chihuahuan propolis was physicochemically and biologically characterized, including instrumental colorimetry (CIELab parameters), gravimetric analysis of moisture, lipid, and ash content, protein measurement, and quantification of total phenols and flavonoids using spectrophotometry with gallic acid and quercetin calibration curves, respectively. Its antioxidant capacity (IC_50_) was assessed through a free radical scavenging assay, and antimicrobial activity was tested against representative bacteria via broth or agar susceptibility testing with *Staphylococcus aureus* (ATCC 25923), *Melissococcus plutonius* (ATCC 35311).

### 2.3. Root Canal Preparation and Sterilization

Soft tissue remnants were removed with an ultrasonic scaler. A low-speed diamond disc (BredentR, Wittighausen, Senden, Germany) mounted on a cutting machine and cooled with water was used to section the crowns between the cementoenamel junction and the apical third of the root, obtaining 6 mm of the middle third of the root.

The canals were prepared with a Peeso No. 2 reamer (Mani^®^, Utsunomiya, Tochigi, Japan) using a low-speed handpiece (Kavo^®^, Charlotte, NC, USA) to standardize the internal diameter of the root canals to 0.9 mm, 0.5 mm from the apical foramen. The smear layer was removed by sonic irrigation (EndoActivator^®^, Dentsply^®^, Weybridge, Surrey, UK) with 5.25% NaOCl (Clorox^®^, Oakland, CA, USA), followed by 17% EDTA (Calasept^®^, Nordiska Dental^®^, Angelholm, Skåne County, Sweden) for 5 min. Afterwards, the dentin specimens were sterilized in a steam autoclave at 121 °C and 15 psi for 20 min. To verify the efficacy of the sterilization procedure prior to inoculation, two randomly selected roots were incubated in Brain Heart Infusion (BHI) broth at 37 °C for 24 h, showing no microbial growth. The confirmed sterile specimens were stored in sterile water at 4 °C under refrigeration until further processing.

### 2.4. Preparation of Propolis Extract and Triple Antibiotic Paste

Chihuahuan propolis was stored in a dark, closed container at −20 °C until processing in order to prevent light induced degradation and unwanted oxidation. The raw propolis was cut into small pieces and ground into powder. The powdered material was mixed with 70% (*v*/*v*) ethanol (Fermont) at a 1:10 (*w*/*v*) ratio for 1 h at room temperature. The mixture was then incubated overnight in the dark and filtered through Whatman No. 1 filter paper (Merck KGaA, Darmstadt, Germany). The filtrate was concentrated in a rotary evaporator under reduced pressure at 39 °C, and the resulting extract was lyophilized. The dried ethanolic extract was stored in a dark container at −20 °C until use. For other methodology assays, the extract was reconstituted with dimethyl sulfoxide (DMSO, Sigma-Aldrich, St. Louis, MO, USA). This initial solution was then brought to volume with sterile saline to achieve a stock concentration of 70 mg/mL. Working solutions of 35 and 15 mg/mL were subsequently prepared by diluting the stock with sterile saline. To ensure the stability of the bioactive constituents and maximize antimicrobial efficacy, all experimental samples were freshly prepared immediately before being used in the assays [2].

### 2.5. Determination of MIC and Disk Diffusion

The MIC of propolis against *E. faecalis* was determined by broth microdilution in 96-well plates using a standardized suspension of 1 × 10^6^ CFU/mL and resazurin as a viability indicator. After 24 h of incubation at 37 °C and an additional 2 h with resazurin (Trek Diagnostic Systems, Inc., Independence, OH, USA), the MIC was defined as the lowest concentration that maintained the blue color. the MIC assay was performed using three technical replicates per concentration across three independent experiments.

For disk diffusion, Mueller–Hinton agar plates were lawn inoculated with the same *E. faecalis* suspension. Sterile paper disks (6 mm) were soaked with 10 µL of each experimental solution and placed on the agar surface. The groups included saline (negative control), amoxicillin clavulanate (positive control), and propolis at 70, 35, and 15 mg/mL. After 24 h at 37 °C, inhibition halos were measured with a Vernier caliper. These assays were performed with three technical replicates per group in three independent experiments.

### 2.6. Root Canal Infection Model

In this study, the experiments were performed using an extracted human tooth model, providing a more realistic simulation of clinical treatment as previously described [12]. A standard *E. faecalis* (ATCC 29212) strain was reactivated in Brain Heart Infusion broth (BD DifcoTM, Franklin Lakes, NJ, USA) and adjusted to 0.5 McFarland units (approximately 1.5 × 10^8^ CFU/mL). Each sterilized canal was inoculated with 10–20 µL of the suspension and gently instrumented with a sterile K file (Dentsply) to evenly distribute the inoculum along the working length. Access cavities were sealed with a sterile provisional material, and samples were incubated at 37 °C for 28 days, with periodic renewal of the medium every 48–72 h to penetrate deeply into dentinal tubules and survive under nutrient-deprived conditions for extended periods. This promotes stable intratubular colonization and mature biofilm formation, which more closely resemble the clinical conditions observed in persistent endodontic infections than short-term planktonic models.

### 2.7. Experimental Groups and Intracanal Medication

Following the 28-day incubation period, stable intratubular colonization was confirmed on selective agar. The total sample of 25 infected dentin specimens was then randomly assigned to five experimental groups (*n* = 5 roots per group) using a computer-generated simple randomization sequence to prevent selection bias. The experimental groups were defined as follows: group 1: saline (negative control); group 2: TAP (positive control); group 3: propolis 70 mg/mL; group 4: propolis 35 mg/mL; group 5: propolis 15 mg/mL. The intracanal medicaments were placed in the canal using a sterile 5.0 mL syringe (UltraDent^®^, South Jordan, UT, USA) and Endo Eze needle until the canal was completely filled. Each root was kept in an incubator at 37 °C for the experimental period of 7 days.

### 2.8. Antimicrobial Evaluation

After treatment, roots were gently rinsed in their own medium with sterile saline, and canals were irrigated with thioglycolate broth as a transport and neutralizing medium. The first sample (T1) was collected using a sterile paper point for 60 s and then transferred to 500 µL of thioglycolate in an Eppendorf tube. The canals were then re-instrumented with a Peeso #2 bur to generate dentin debris, and a second paper point sample (T2) was obtained and placed in another 500 µL of thioglycolate. After vortexing, 20 µL aliquots of each suspension and a 1:10 dilution of T2 were plated on bile esculin agar. For this assay, each infected tooth (*n* = 5 per experimental group) was considered an independent biological replicate. To ensure accuracy in the viable bacterial counts, all sample dilutions were plated in triplicate. The plates were then incubated at 37 °C for 24 h. CFU/mL were counted and expressed as mean ± standard deviation, and log_10_ reductions were calculated relative to the negative control.

### 2.9. Scanning Electron Microscopy (SEM)

Root canal samples (*n* = 5 per group) were fixed in 2–2.5% glutaraldehyde, washed with phosphate-buffered saline, dehydrated in ascending grades of ethanol for 20 min each, and then immediately transferred into the pressure chamber of a critical point drying machine. The specimens were mounted on aluminum stubs with double-sided conductive tape, and a 30 nm thick gold layer was sputtered onto them for two minutes. Afterward, the root canals were examined using a phase analysis performed with backscattered electron imaging on a Hitachi SU3500 scanning electron microscope (Nanotech Laboratory, Singapore).

### 2.10. Statistical Analysis

All quantitative data were expressed as mean ± standard deviation (SD). The normality of the data distribution was first evaluated using the Shapiro–Wilk test. For the planktonic assays (MIC determination and disk diffusion), differences between groups were analyzed using a one-way analysis of variance (ANOVA) followed by Tukey’s post hoc test. For the intratubular infection model, the raw colony-forming unit (CFU/mL) counts were log_10_-transformed prior to analysis to ensure constant variance and satisfy parametric assumptions. The log_10_ reduction values were subsequently analyzed using a one-way ANOVA with Tukey’s multiple comparisons test to evaluate differences between the experimental medicaments and the control groups. In cases where the data did not meet parametric assumptions, the Kruskal–Wallis test followed by Dunn’s post hoc test was applied. For all statistical analyses, the significance level was strictly set at alpha (*p* < 0.05). All statistical procedures and graphing were performed using GraphPad Prism version 9.0 (GraphPad Software, San Diego, CA, USA).

## 3. Results

### 3.1. Physicochemical and Biological Characterization of Propolis

The physicochemical propolis from Cuauhtémoc, Chihuahua, showed a yellowish-green color (L* 54.85, b* 18.20), lipid content (32.2 ± 1.4%), protein content (2.13 ± 0.5%), and ash content (2.48 ± 0.5%). Total phenols ranged from 193.12 ± 25.5 mg GAE/mL and flavonoids from 33.36 ± 4.5 QE/mL, with antioxidant IC_50_ values between 296 ± 15.5 µg/mL. Moreover, the propolis showed antimicrobial activity against *Staphylococcus aureus* and *Melissococcus plutonius.* Detailed Physicochemical and biological characterization is presented in Table 1.

### 3.2. Antimicrobial Activity of the Propolis Extract

The MIC of the propolis extract against *E. faecalis* was 17.5 mg/mL, within a tested range of 70–1.09 mg/mL, while the TAP control inhibited growth at ≤1.0 mg/mL and the negative control showed positive growth (Table 2).

The disk diffusion assay results showed a clear dose-dependent antimicrobial effect of propolis. The saline solution used as the negative control exhibited no inhibitory activity, with an inhibition zone of 0.0 mm. In contrast, the positive control (amoxicillin + clavulanic acid) produced the largest inhibition zone (24.6 ± 1.2 mm), confirming the assay’s validity. Propolis at 70 mg/mL demonstrated substantial antimicrobial activity, with a mean inhibition zone of 18.4 ± 1.0 mm. A progressive reduction in inhibitory effect was observed as the concentration decreased, with inhibition zones of 13.2 ± 0.9 mm and 8.1 ± 0.7 mm for propolis at 35 mg/mL and 15 mg/mL, respectively. These findings indicate a concentration-dependent antimicrobial response of propolis, although its activity remained lower than that of the positive control across all tested concentrations (Table 3).

### 3.3. Intracanal Antimicrobial Activity in Dentin

After 7 days of intracanal medication, a significant reduction in bacterial load was observed, depending on the treatment used. The negative control group showed the highest bacterial counts, with a mean of (3.2 ± 0.4) × 10^6^ CFU/mL, and no log_10_ reduction, confirming ongoing bacterial growth without an antimicrobial agent. In contrast, the positive control demonstrated a strong antimicrobial effect, lowering bacterial counts to (8.5 ± 3.2) × 10^1^ CFU/mL, which corresponds to a 4.6 log_10_ reduction. Propolis at 70 mg/mL showed similar antibacterial activity, with bacterial counts of (1.2 ± 0.5) × 10^2^ CFU/mL and a log_10_ reduction of 4.4, indicating potent intracutaneous antimicrobial action. A concentration-dependent decrease in effectiveness was noted for lower propolis concentrations. Treatment with propolis at 35 mg/mL resulted in (4.1 ± 0.8) × 10^3^ CFU/mL and a 2.9 log_10_ reduction, while the lowest concentration (15 mg/mL) produced (9.6 ± 1.1) × 10^4^ CFU/mL with a 1.5 log_10_ reduction. Overall, these results show a clear dose-dependent antibacterial effect of propolis, with the highest concentration nearing the efficacy of the positive control (Table 4).

### 3.4. SEM Observations

SEM analysis of the root canal walls and dentinal tubules showed significant differences among the experimental groups. In the negative control group, extensive bacterial colonization was observed, characterized by dense clusters of coccoid-shaped microorganisms that looked like *E. faecalis*. These bacteria mainly adhered to the entrances of the dentinal tubules (A) and were also seen penetrating and extending along the tubule tracts (B), indicating deep intratubular invasion and biofilm formation. In contrast, specimens treated with positive control (triple antibiotic paste, TAP) showed a strong antimicrobial effect. Both the dentinal tubule entrances and their intratubular pathways were mostly free of coccoid bacterial structures, with clean canal walls and no signs of bacterial adhesion or biofilm remnants, demonstrating effective removal of *E. faecalis* from the dentin surface and tubule interiors.

**Figure 1:**
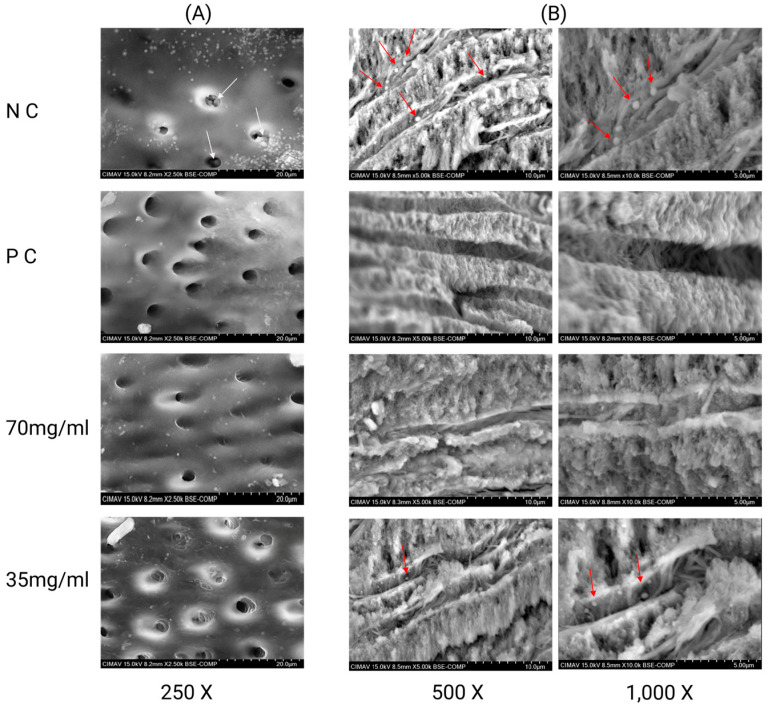
Scanning electron microscopy (SEM) images of root canal walls and dentinal tubules after intracanal treatments. (**A**) Representative images of dentinal tubule entrances and (**B**) higher-magnification views of dentinal tubule pathways. Scale bars: 20 µm for dentinal tubule entrances; 10 µm and 5 µm for dentinal tubule pathways at higher magnifications. The images correspond to the following experimental groups: NC (negative control, saline solution), PC (positive control), and propolis-treated groups at concentrations of 70 and 35 mg/mL. Arrows (red or white) indicate coccoid bacterial cells that are morphologically similar to *E. faecalis*.

The group treated with propolis at a concentration of 70 mg/mL showed a significant reduction in bacterial presence. SEM micrographs revealed dentinal tubules free of bacterial cells and organic debris, with well-defined tubule outlines and intact dentin structure. In contrast, samples treated with propolis at 35 mg/mL displayed a partial antimicrobial effect. Although there was a clear decrease in bacterial density compared to the negative control, some coccoid cells remained visible in certain tubules and in the superficial parts of others. Additionally, the remaining bacterial cells showed mild morphological changes, such as surface irregularities and apparent structural damage, which may suggest sublethal effects of the propolis extract at this lower concentration (Figure 1).

## 4. Discussion

The current study showed that Chihuahuan propolis has strong antimicrobial activity against *E. faecalis* inside infected dentinal tubules. Notably, propolis at 70 mg/mL lowered the bacterial load within the tubules to levels like those achieved with TAP. This is especially important given the rising concerns about the routine use of antibiotic-based intracanal medicaments and their drawbacks, such as tooth discoloration, cytotoxicity, and contribution to antimicrobial resistance. These results are aligned with previous reports indicating that propolis has demonstrated significant intracanal effectiveness against *E. faecalis* and other endodontic pathogens, alone or in combination with other agents [11,12]. However, most of those studies have focused on comparisons with calcium hydroxide or chlorhexidine rather than TAP as an antibiotic benchmark. In this context, the present work provides additional evidence that a region-specific propolis, with a unique phenolic and flavonoid profile, can approach the antimicrobial performance of TAP in a persistent dentin infection model, reinforcing its potential as a natural alternative when long-term intracanal antibiotic use is not desired.

The intratubular infection model employed in this study, based on prolonged incubation to promote mature bacterial colonization, enhances the clinical relevance of the findings, as *E. faecalis* is known to persist within dentinal tubules in cases of endodontic failure. The SEM observations further supported the microbiological results by demonstrating reduced bacterial presence within dentinal tubules following propolis application.

Previous literature has reported heterogeneous results regarding the antibacterial potency of propolis, ranging from moderate effects against *E. faecalis* to efficacy comparable or superior to various intracanal medicaments, depending on the vehicle, concentration, and exposure time employed [11,13,14]. In a recent study, propolis formulated as an intracanal medicament demonstrated significant antibacterial activity against *E. faecalis* in primary teeth, although its performance was mainly evaluated against calcium hydroxide [15]. Other in vitro and clinical investigations have suggested that propolis, alone or combined with agents such as moxifloxacin, can reduce bacterial loads to levels similar to those achieved with TAP in teeth with apical periodontitis, although intergroup differences are not always statistically significant [16,17]. The finding that Chihuahuan propolis at 70 mg/mL produced a 4.4 log_10_ reduction in CFU/mL is comparable to the antimicrobial efficacy of TAP, which can be largely explained by the diffusion dynamics within the complex dentinal tubule network. According to Fick’s first law of diffusion, a higher initial concentration of a medicament creates a steeper concentration gradient, which acts as the primary driving force for deep intratubular penetration [18]. In this sense, the lower concentrations (15 and 35 mg/mL), the diffusion of propolis molecules is likely exhausted before reaching the deep-seated *E. faecalis* biofilms. However, at 70 mg/mL, the abundant low-molecular-weight bioactive compounds such as phenolic acids and flavonoids achieve sufficient mass transfer to penetrate deeply into the dentin, mirroring the high penetrability of the small antibiotic molecules present in TAP. Furthermore, the use of a highly penetrative vehicle like DMSO to solubilize the extract likely reduced the surface tension of the medicament, enhancing dentin wettability and facilitating the optimal transport of these polyphenols into the narrow and complex microanatomy of the root canal system.

Moreover, recent in vitro and ex vivo studies have demonstrated that propolis extracts, whether used independently or mixed with calcium hydroxide, exhibit potent antibiofilm efficacy comparable or even superior to standard calcium hydroxide pastes [19]. Furthermore, the endodontic field is also investigating natural alternatives; recent studies show that agents like Aloe vera and chitosan with mangosteen extracts offer strong, biocompatible antibacterial effects against *E. faecalis* in the root canal system [20]. Our results contribute to this contemporary paradigm by validating the specific efficacy of Chihuahuan propolis in a mature intratubular biofilm model.

In the current context of rising antimicrobial resistance and growing concerns about adverse effects associated with TAP, these results gain additional clinical significance [21,22]. TAP, composed of metronidazole, ciprofloxacin, and minocycline, has shown high effectiveness against endodontic pathogens but has also been associated with tooth discoloration, reduced dentin microhardness, and potential development of resistance due to prolonged use of broad-spectrum antibiotics [23]. Conversely, propolis is generally considered less cytotoxic than calcium hydroxide and well-tolerated, with few reports of hypersensitivity. It also exhibits antioxidant and immunomodulatory properties that may promote periapical tissue healing [24,25,26]. Propolis could serve as an intracanal medicament when TAP is contraindicated, or resistance minimization is prioritized due to its strong antimicrobial activity and favorable biological profile [27,28].

In this study, the MIC of 17.5 mg/mL against *E. faecalis* was higher than that of TAP (≤1.0 mg/mL), indicating a lower intrinsic potency of the extract under broth conditions; however, this difference was offset in situ by using a high intracanal concentration (70 mg/mL). A similar discrepancy between MIC and intratubular efficacy has been reported for other propolis preparations, in which high local concentrations and extended exposure times allow overcoming lower intrinsic potency relative to conventional antibiotics [29,30,31]. The clear dose-dependent response observed here, with 2.9 and 1.5 log_10_ reductions at 35 and 15 mg/mL, respectively, underscores that the therapeutic window of propolis as an intracanal medicament may critically depend on achieving sufficiently high concentrations and maintaining adequate contact times, with direct implications for the design of clinical formulations and application protocols.

The SEM findings provide a valuable morphological complement to microbiological data. The presence of abundant coccoid cells compatible with *E. faecalis* on dentin surfaces and within dentinal tubules in the negative control group reproduces the classic picture of a mature, highly resistant intratubular biofilm. In contrast, both TAP and propolis at 70 mg/mL resulted in dentinal tubules virtually free of coccoid structures, with clean canal walls and no recognizable bacterial aggregates, whereas the intermediate concentration of 35 mg/mL produced only partial cleaning and residual bacteria showing mild morphological changes. Similar SEM observations have been reported for chitosan–propolis nanoparticles and other disinfection protocols, in which disruption of biofilm structure and membrane damage are key antimicrobial mechanisms [32,33,34]. These data support the idea that Chihuahuan propolis not only reduces bacterial counts but also affects biofilm organization and intratubular colonization, which are critical for preventing infection relapse.

The 28-day *E. faecalis* infection model used demonstrates a strong experimental setup that mimics mature intratubular biofilms. It shows that propolis at 70 mg/mL resulted in a 4.4 log_10_ bacterial reduction—almost the same as TAP’s 4.6 log_10_—defining a clear therapeutic concentration range (4.4, 2.9, 1.5 log_10_ at 70, 35, 15 mg/mL). Extensive physicochemical analysis (phenolics: 193–214 mg GAE/mL; flavonoids: 33–36 mg QE/mL) and SEM-confirmed biofilm disruption highlight propolis as a promising intracanal medicament. It offers translational benefits over TAP, including a lower risk of discoloration, preservation of dentin qualities, and antimicrobial stewardship advantages. Future research should focus on multispecies biofilms, combination therapies with sonic activation, standardized 70 mg/mL formulations, and clinical trials to assess healing outcomes.

This study acknowledges several inherent limitations. First, while our 28-day intratubular incubation protocol successfully promotes deep dentinal colonization and monospecific biofilm-like structures, we recognize that this in vitro model does not fully replicate the highly complex, multispecies biofilm matrix found in true clinical endodontic infections. In vivo biofilms possess a robust extracellular polymeric substance that significantly increases resistance to intracanal medicaments compared to mono-species models. Second, propolis is a natural product whose chemical composition varies based on geographic origin, local flora, and extraction methods. Although our study confirmed its rich polyphenolic profile, this inherent variability remains a challenge for clinical standardization. Nevertheless, compared to antibiotic-based medicaments like TAP, propolis offers the critical advantage of mitigating the risk of antimicrobial resistance, a major global health concern. Furthermore, it circumvents adverse effects commonly associated with TAP, such as tooth discoloration and dentin demineralization. To overcome the limitations of natural variability, future research must focus on advanced chemical fingerprinting techniques, followed by rigorous in vivo and clinical trials to establish standardized, safe, and reproducible propolis-based intracanal therapies.

Finally, Chihuahuan propolis should be regarded as a promising candidate for incorporation into intracanal disinfection regimens, particularly in settings where reducing systemic and topical antibiotic use is a therapeutic priority.

## 5. Conclusions

Chihuahuan propolis at 70 mg/mL shows antimicrobial activity in the intratubular space similar to that of TAP against mature *E. faecalis* biofilms, offering a promising natural alternative for intracanal disinfection. From a translational perspective, the current findings support further research on Chihuahuan propolis as a potential intracanal medicament. However, additional studies are necessary to assess its cytotoxicity to periapical tissues, effects on dentin structure, and antimicrobial activity in multispecies biofilm models. Such assessments are crucial to determine the safety, optimal formulation, and clinical potential of propolis-based intracanal treatments before clinical use.

## Figures and Tables

**Table 1:** Physicochemical and biological characterization of propolis.

Parameter	Result
Color	Yellowish green; in some samples, darker green (L* = 54.85, b* = 18.20)
Lipids	32.2 ± 1.4%
Proteins	2.13 ± 0.5%
Ash	2.48 ± 0.5%
Total phenolic content	193.12 ± 25.5 mg GAE/mL
Total flavonoid content	33.36 ± 4.5 mg QE/mL
Antioxidant activity (IC_50_)	296 ± 15.5 µg/mL
Antimicrobial activity	*Staphylococcus aureus* *Melissococcus plutonius*

Values are expressed as mean ± standard deviation (SD) of three independent experiments performed in triplicate.

**Table 2:** Minimum inhibitory concentration of the propolis extract.

Treatment	Range of Concentrations Evaluated (mg/mL)	MIC (mg/mL)
Negative control	—	Positive growth
Positive control	—	≤1.0
Propolis	70–1.09	17.5

Values are expressed as mean ± standard deviation (SD) of three independent experiments performed in triplicate.

**Table 3:** Disk diffusion assay.

Group	Treatment	Inhibition Zone (mm) Mean ± SD
Group 1	Negative control	0.0 ± 0.0
Group 1	Positive control	24.6 ± 1.2
Group 2	Propolis 70 mg/mL	18.4 ± 1.0
Group 3	Propolis 35 mg/mL	13.2 ± 0.9
Group 4	Propolis 15 mg/mL	8.1 ± 0.7

Negative control (Saline solution), Positive control (Amoxicillin + clavulanic acid). Values are expressed as mean ± standard deviation (SD) of three independent experiments performed in triplicate (*N* = 3).

**Table 4:** Intracanal antimicrobial activity in dentin.

Group	Intracanal Treatment	CFU/mL (Mean ± SD)	Log_10_ CFU Reduction *	*p* Value
Group 1	Negative control	(3.2 ± 0.4) × 10^6^	0.0	---
Group 2	Positive control	(8.5 ± 3.2) × 10^1^	4.6	** p < 0.001
Group 3	Propolis 70 mg/mL	(1.2 ± 0.5) × 10^2^	4.4	** p < 0.001
Group 4	Propolis 35 mg/mL	(4.1 ± 0.8) × 10^3^	2.9	** p < 0.001
Group 5	Propolis 15 mg/mL	(9.6 ± 1.1) × 10^4^	1.5	* p < 0.05

Intracanal antimicrobial activity against *E. faecalis* in dentin after 7 days of medication. Values represent mean ± standard deviation (SD) from five biological replicates (n = 5). * Log_10_ reduction calculated relative to negative control. One-way ANOVA with Tukey’s post hoc test: ** *p* < 0.001 vs. negative control.

## Data Availability

The original contributions presented in this study are included in the article. Further inquiries can be directed to the corresponding authors.

## Abbreviations

CFU	Colony-Forming Units
MIC	Minimum Inhibitory Concentration
SEM	Scanning Electron Microscopy
EDTA	Ethylenediaminetetraacetic acid
NaOCl	Sodium Hypochlorite
TAP	Triple Antibiotic Paste
ATCC	American Type Culture Collection
SD	Standard Deviation
ANOVA	Analysis of Variance
GAE	Gallic Acid Equivalents
QE	Quercetin Equivalents
IC_50_	Half Maximal Inhibitory Concentration
DMSO	Dimethyl Sulfoxide
